# Combined 17β-Estradiol with TCDD Promotes M2 Polarization of Macrophages in the Endometriotic Milieu with Aid of the Interaction between Endometrial Stromal Cells and Macrophages

**DOI:** 10.1371/journal.pone.0125559

**Published:** 2015-05-07

**Authors:** Yun Wang, Hong Chen, NingLing Wang, HaiYan Guo, Yonglun Fu, Songguo Xue, Ai Ai, Qifeng Lyu, Yanping Kuang

**Affiliations:** Department of Assisted Reproduction, Shanghai ninth people’s hospital affiliated to JiaoTong University School of Medicine, Shanghai, China; Nihon University School of Medicine, JAPAN

## Abstract

The goal of this study is to elucidate the effects of 17β-estradiol and TCDD (2,3,7,8-tetrachlorodibenzo-p-dioxin) on macrophage phenotypes in the endometriotic milieu. Co-culture of endometrial stromal cells (ESCs) and U937 cells (macrophage cell line) was performed to simulate the endometriotic milieu and to determine the effects of 17β-estradiol and/or TCDD on IL10, IL12 production and HLA-DR, CD86 expression by U937 macrophages. We found that combining 17β-estradiol with TCDD has a synergistic effect on inducing M2 activation when macrophages are co-cultured with ESCs. Moreover, the combination of 17β-estradiol and TCDD significantly enhanced STAT3 and P38 phosphorylation in macrophages. Differentiation of M2 macrophages induced by 17β-estradiol and TCDD were effectively abrogated by STAT3 and P38MAPK inhibitors, but not by ERK1/2 and JNK inhibitors. In conclusion, 17β-estradiol and TCDD in the ectopic milieu may lead to the development of endometriosis by inducing M2 polarization of macrophages through activation of the STAT3 and P38MAPK pathways.

## Introduction

Endometriosis, a chronic inflammatory disease, is a gynecological disorder which has a complex, multifactorial etiology, ultimately leading to severe pelvic pain and, in some cases, infertility. A leading theory as to its etiology is that endometriosis is caused by retrograde displacement of eutopic endometrium into the pelvis and its subsequent implantation on peritoneal surfaces. A lack of adequate immune surveillance in the peritoneum is thought to contribute as well. Peritoneal macrophages isolated from patients with endometriosis were found to have poor phagocytic capacity; this and other phenotypic and functional alterations were associated with disease severity[1,2]. Our previous work has indicated that macrophages are involved in ectopic adhesion, implantation, and growth of the endometriotic tissue, as opposed to clearing[3–7].

Macrophages can be subdivided into classically activated macrophages (M1 macrophages) and alternatively activated macrophages (M2 macrophages). M1 macrophages are potent effector cells that kill microorganisms and produce pro-inflammatory cytokines, such as tumor necrosis factor-α (TNF-α), IL-6, and IL-12. In contrast, M2 macrophages reduce these inflammatory Th1 responses by producing anti-inflammatory factors (IL-10, TGF-β and IL-1 receptor antagonist), and promote angiogenesis, tissue remodeling, and repair. Macrophages are plastic cells, and can switch from an activated M1 state back to M2, and vice versa, upon receiving specific signals[8]. M1 macrophages have low expression of CD14 and high expression of HLA-DR and CD86 on their surface, while M2 macrophages have increased expression of CD14 and decreased expression of HLA-DR and CD86, which enables the identification of M2 macrophages by their altered cell surface phenotypes[9].

In humans, the increased incidence of endometriosis has been associated with exposure to environmental chemicals[10]. TCDD (2,3,7,8-tetrachlorodibenzo-*p*-dioxin), a member of the dioxin family of chlorinated aromatic hydrocarbons, is ubiquitous and one of the most feared environmental contaminants worldwide. TCDD is derived from sources of 2,4,5-trichlorophenol[11–12]. The myriad of biological effects of TCDD is believed to be mediated via the aryl hydrocarbon receptor (AhR)[13], which forms a complex with the AhR nuclear translocator (ARNT) to activate TCDD responses[11,14–16]. TCDD exposure has been shown to induce both cytochromes P450 1A1(CYP 1A1)[17–18] and CYP1B1[19] in various tissues. Both CYP1A1 and CYP1B1 are 17β-estradiol hydroxylases [20–21]. The TCDD/AhR/ARNT heteromeric complex acts as a signal transducer and transcription factor for target genes, including CYP1A1, CYP1A2 and CYP1B1, and genes involved in cell proliferation, differentiation and inflammation[22]. Since endometriosis is an estrogen- dependent disease[23–26], and the inflammatory milieu in the peritoneal cavity of women with endometriosis has been extensively characterized, we wondered if altered metabolism of estradiol by TCDD or other dioxin-like halogenated aromatic hydrocarbons and pro-inflammatory effects of TCDD may be involved in disease pathogenesis. Our previous work demonstrated that the combination of 17β-estradiol and TCDD upregulates CXCR1 and CCR8 expressions in ESCs, and promotes the secretion of their respective ligands, chemokine IL8 and I-309, in co-cultures of endometriotic focus-associated cells [3–4].

In this study, we first evaluated macrophage IL10 and IL12 secretion and CD86 and HLA-DR expression by cell co-culture. Next, we studied the effects of 17β-estradiol and/or TCDD on macrophages phenotype in the co-culture system. Lastly, we investigated the intracellular signaling pathways involved in 17β-estradiol and TCDD-induced M2 macrophage differentiation.

## Materials and Methods

### Tissue collection, cell isolation and culture

Samples of endometriotic peritoneal surface lesions (n = 8) and ovarian lesions (n = 8) were obtained from women (age range 26–44) undergoing laparoscopy for pain or other benign indications. The patients with endometriosis were classified according to the revised American Fertility Society (AFS) classification: 7 women were classified as stage 1 and 9 as stage 2. None of the women had received hormonal medication in the 3 months prior to the surgical procedure. All the samples were obtained in the proliferative phase of the cycle, which was confirmed histologically according to established criteria. All procedures involving participants in the study were approved by the Human Research Ethics Committee of Shanghai Ninth People’s Hospital affiliated to Jiaotong University School of Medicine, and all subjects signed a written consent for the collection of tissue samples.

The endometrial tissues were collected under sterile conditions and transported to the laboratory on ice in DMEM (Dulbecco’s modified Eagle’s medium)/F-12 (Gibco, USA) supplemented with 10% fetal calf serum (FCS; Hyclone, Logan, UT, USA). ESCs were isolated according to previously-described methods[27]. Briefly, endometrial tissues were digested with collagenase type IV (0.1%; Sigma, USA) for 30 min at 37°C with constant agitation. The tissue pieces were filtered through sterile gauze pads (pore diameter sizes:200 mesh) to remove cellular debris. Following gentle centrifugation, the supernatant was discarded and the cells were resuspended in DMEM/F-12. The ESCs were separated from epithelial cells by passing them over sterile gauze pads (pore diameter sizes: 400 mesh). The filtrated suspension was layered over Ficoll and centrifuged at 800×g for 20 min to further remove leukocytes and erythrocytes, and the middle layer was collected and then washed with D-Hanks solution. ESCs were placed in a culture flask and allowed to adhere for 20 min. The adherent stromal cells were cultured as a monolayer in flasks with DMEM/F-12 supplemented with 10% FCS and 20 mmol/l HEPES and incubated in a humidified incubator with 5% CO2 at 37°C. This method supplied 95% vimentin-positive and cytokeratin-negative ESCs.

Human macrophage U937 cells (Cell Bank, Chinese Academy of Sciences, Shanghai, China) were maintained in Roswell Park Memorial Institute (RPMI) 1640 medium (Life Technologies) with 10% bovine calf serum and containing 20 mmol/l HEPES, 100 IU/ml penicillin, and 100 mg/ml streptomycin at 37°C in a humidified, 5% CO2 incubator. The medium was changed every other day.

### Co-culture unit of ESCs and U937 cells

Freshly isolated ESCs were seeded at a density of 1×10^5^ cells per well in 24-well plates overnight. The supernatants were discarded, and the same number of U937 cells were added into each well. The cells were cultured in a final volume of 1 mL of fresh DMEM/F-12 with 2.5% FCS for 48 hours. The U937 cells cultured alone (without ESCs) in the same media served as controls. Each experiment was repeated three times using ESCs obtained from three different patients.

### Treatment in vitro with estrogen and/or TCDD

After serum starvation for 12 hours, ESCs, U937 cells, and co-cultures were treated with different concentrations of 17β-estradiol (0.01, 0.1, 1, 10, and 100 nM) (Sigma, USA) or TCDD (0.01, 0.1, 1, and 10 nM) (Sigma, USA), or the combination of 1nM 17β-estradiol and 1nM TCDD for 48h with vehicle (DMSO) serving as controls. Each experiment was carried out in triplicate, and repeated three times.

### ELISA

Culture supernatants from U937 cells grown alone and ESCs-U937 co-cultures were harvested, centrifuged at 2000×g, then moved to a new tube and stored at -80°C until assayed by ELISA. Each experiment was carried out in triplicate and repeated three times. IL10 and IL12 concentrations were quantified by ELISA (R&D Systems) according to the manufacturer’s instructions.

### Flow cytometry analysis

U937 cells grow in suspension and ESCs are adherent. After U937 cells were co-cultured with ESCs for 48–72h, suspension cells were collected and stimulated with LPS (10 ng/ml) for 24 h. The cells were then incubated for 30 minutes at room temperature with 80 ul of PBS containing 0.2% BSA (PBS–BSA) supplemented with 20 ul PE-conjugated anti-human CD86, and FITC-conjugated HLA-DR antibody (eBioscience, San Diego, CA, USA). Finally, stained cells were washed with PBS–BSA, and were analyzed by a FACS Calibur flow cytometer (Becton Dickinson, USA).

### Treatment with STAT3, ERK1/2, JNK, or p38MAPK Pathway inhibitors

ESCs and U937 cells were co-cultured as described previously, and 20uM STAT3 pathway inhibitor D4071(Sigma, St. Louis, MO, USA), 20uM ERK1/2 pathway inhibitor U0126 (Calbiochem, San Diego, CA, USA), 20uM JNK pathway inhibitor SP600125 or 20uM p38MAPK pathway inhibitor SB203580 (both from Sigma, St. Louis, MO, USA) were added to the culture supernatants. After 30min, 1nM 17β-estradiol and 1nM TCDD were added to the cells, with PBS serving as the control. 48 hours later, western blot was performed to analyze the levels of the phosphorylated forms and total amounts of STAT3 and P38 in U937 cells.

### Western blots

Cells were lysed in RIPA lysis buffer containing proteinase inhibitors. Each sample (25 ug) was loaded on a 10% SDS-PAGE gel. After electrophoresis, proteins were transferred onto a polyvinyl difluoride membrane (Bio-Rad, Hercules, CA, USA), and membranes were incubated in blocking buffer containing 5% non-fat dry milk for 1 hour at room temperature. Next, membranes were probed overnight at 4°C with a primary antibody in blocking buffer (1:1000 dilution of anti-phospho-STAT3 and anti-STAT3 mAbs, anti-phospho-P38 and anti-P38MAPK mAbs, anti-phospho-ERK1/2 and anti-ERK1/2 mAbs, anti-phospho-JNK and anti-JNK mAbs) (Cell Signal Technology, Danvers, MA, USA), followed by probing with anti-mouse Ig-HRP conjugates (Amersham Biosciences, Buckinghamshire, UK) in blocking buffer at a dilution of 1:3000 for 2 hours at room temperature. Signals were recorded on HyperFilm MP (Amersham Pharmacia Biotech) and developed in a Kodak X-Omat film developer. Results were scanned and densitometrically analyzed using Scion Image software (Scion Corporation, Frederick, MD, USA).

### Statistical Analysis

All values are shown as the mean±SD. Data were analyzed by using one-way analysis of variance and least significant difference (equal variances assumed), or Tamhane’s test (equal variances not assumed) was used post hoc for multiple comparisons utilizing the Statistical Package for the Social Sciences software version 11.5. Differences were considered statistically significant at a P<0.05.

## Results

### Effect of the cell co-culture on macrophage activation

**Fig 1:**
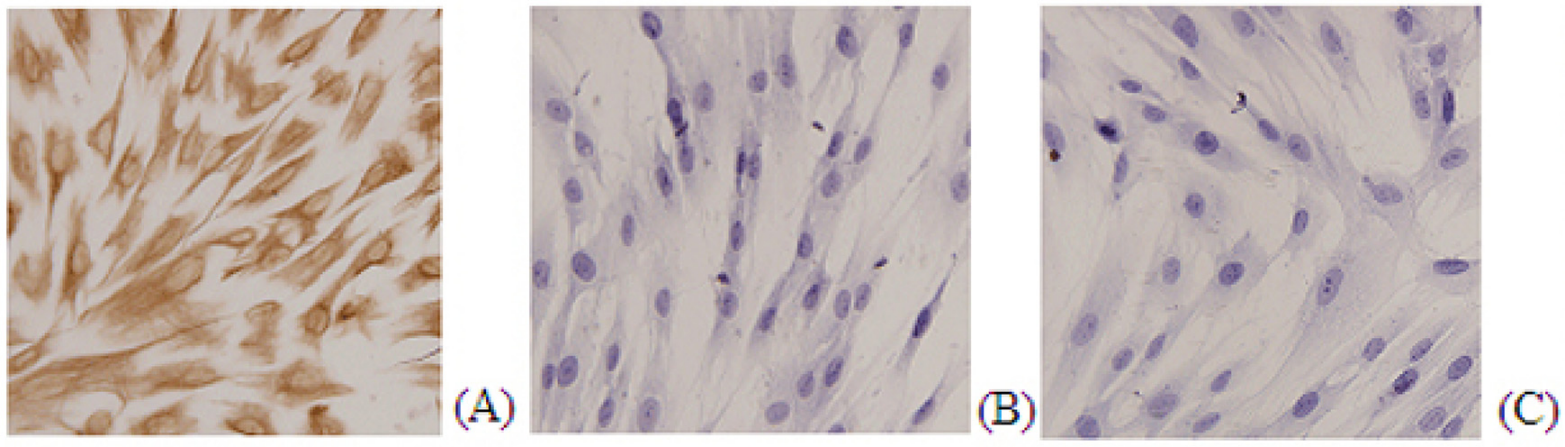
Immunostaining for vimentin (A) cytokeratin (B) and negative control (C) on ESC primary culture.

We evaluated expression of vimentin and cytokeratin on the primary ESC. As shown in **Fig 1A**, almost all ESC stained for vimentin. In contrast, (**Fig 1B)** ESCs were negative for cytokeratin staining.

**Fig 2:**
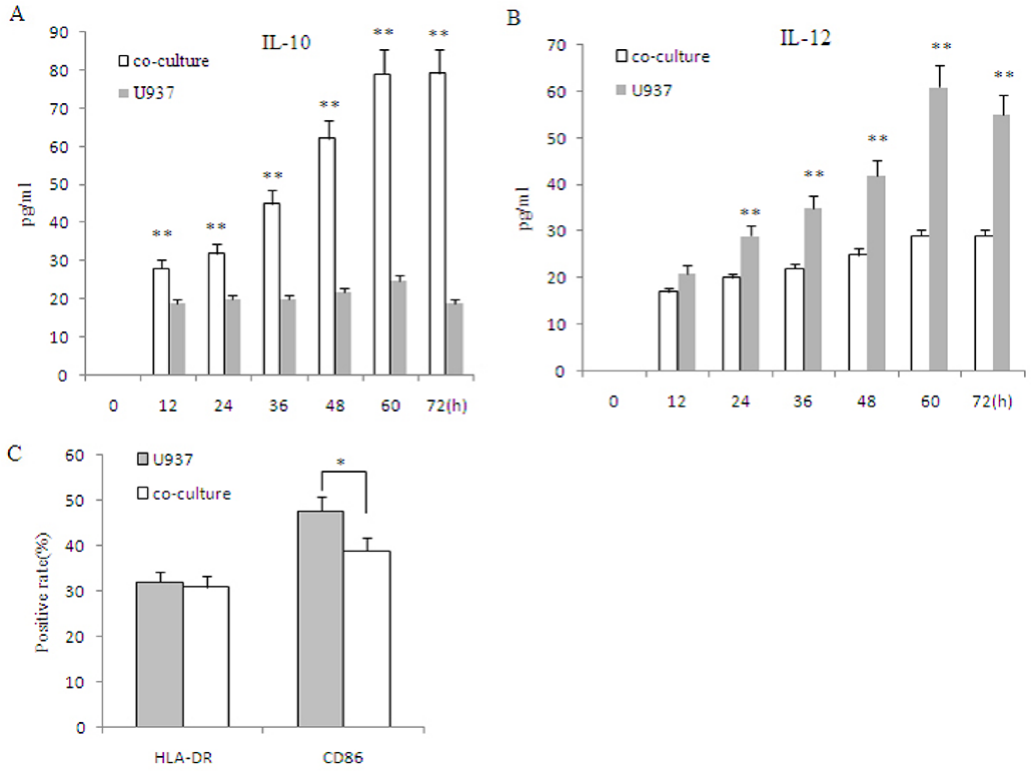
Effect of co-culture on inducing macrophages toward the M2 phenotype. U937 (1×10^5^ cells per well of 24 well plates) was stimulated with LPS (10ng/ml) for 24h after undergoing co-culture with eutopic ESCs for 72h, followed by an analysis of the secretion of IL-10 (A) and IL-12 (B) by ELISA. Surface expression of HLA-DR and CD86 in U937 cells was determined by flow cytometry (C). Eutopic ESCs = ESCs from eutopic endometrium with endometriosis. Data are expressed as mean±SD of independent experiment, performed in triplicate wells with three different samples. *P<0.05 compared with the U937 control, **P<0.01 compared with the U937 control.

Neither IL-10 nor IL-12 secretion was detected in primary ESCs, even after 72 h of culture. To investigate the effects of ESC/macrophage interaction on macrophage polarization, U937 cells were co-cultured with ESC for 72h, and were then stimulated with LPS (10 ng/ml) for 24h. Co-culture increased IL10 secretion, a M2 phenotype marker (*P*<0.01;**Fig 2A**); whereas IL12 secretion, a M1 phenotype marker, was down-regulated compared to control cells. Moreover, co-culture decreased U937 cell CD86 expression compared to control (P<0.05; **Fig 2C**). These results indicate that ESC-U937 co-culture induces macrophage activation towards the M2 phenotype.

### Effect of 17β-estradiol on macrophage activation by co-culture of ESC and U937

**Fig 3:**
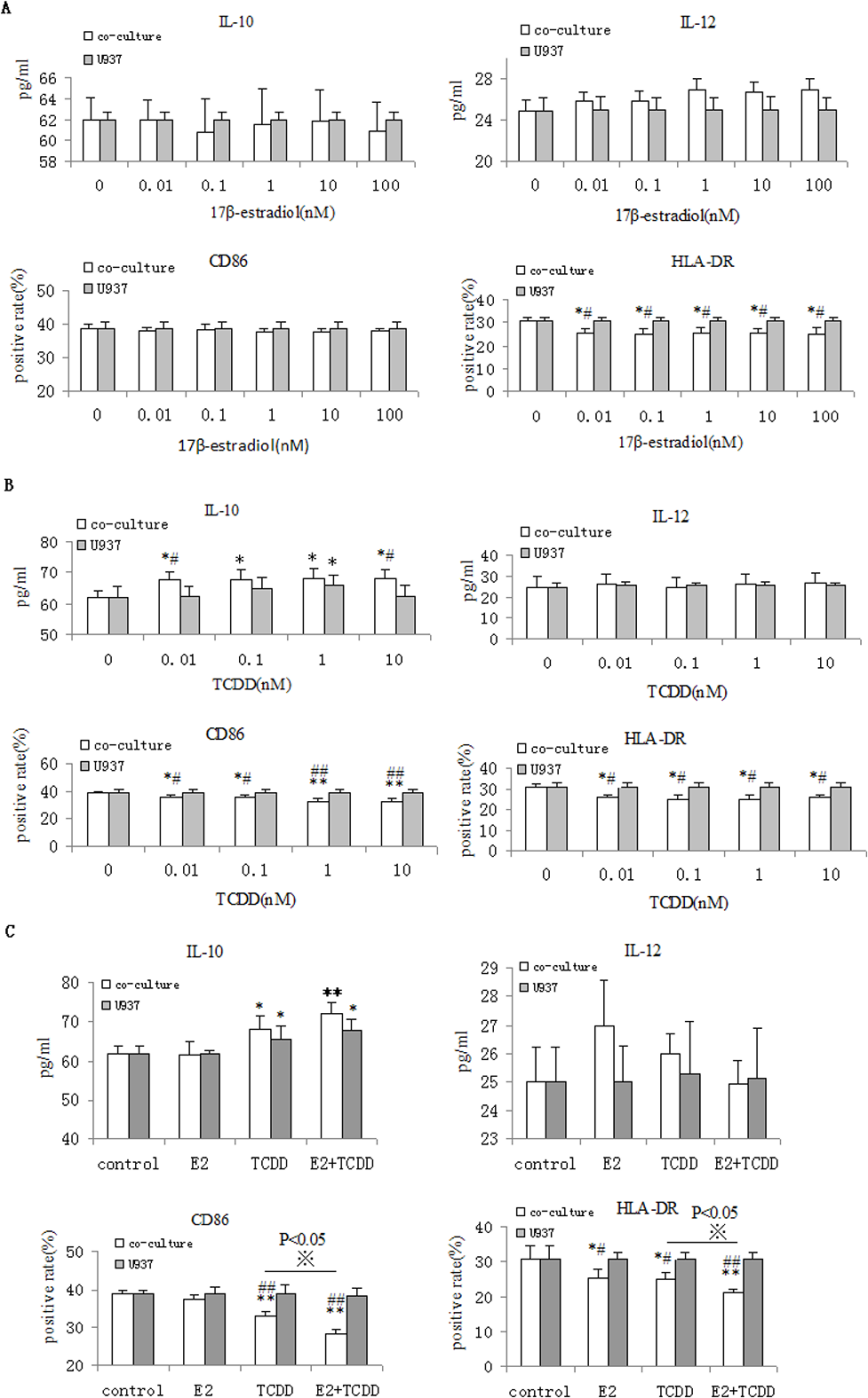
Combination of 17β-estradiol and TCDD promotes M2 macrophage polarization by co-culture of ESC and U937. Co-cultured cells were treated with various concentrations of 17β- estradiol (0.01–100nM) (A) or TCDD(0.1-10nM) (B), or 17β-estradiol (1nM) plus TCDD 1nM) (C) for 48 hours, and then stimulated with LPS (10 ng/ml) for 24h. Finally, IL-10 and IL-12 secretion was measured by ELISA. Surface expression of CD86 and HLA-DR in U937 cells was determined by flow cytometry. Eutopic ESCs = ESCs from eutopic endometrium with endometriosis. Data are expressed as mean±SD of independent experiment, performed in triplicate wells with three different samples. *P<0.05, **P<0.01 compared with the vehicle control. #P<0.05, ##P<0.01compared with the U937 control. ※P<0.05 compared with the TCDD treatment.

0.01–100nM of 17β-estradiol had no obvious effects on IL10 secretion by macrophages (p>0.05;**Fig 3A**). Moreover, 17β-estradiol did not affect IL12 secretion or CD86 expression by U937 cells (p>0.05;**Fig 3A**). 0.01–100nM 17β-estradiol decreased HLA-DR expression in U937 co-cultured with ESCs (p<0.05;**Fig 3A**).

### Effect of TCDD on macrophage activation by co-culture of ESC and U937

TCDD significantly stimulated U937 cells to secrete IL-10 at a dose of 1 nM (*P<0*.05). Morever, TCDD increased U937 cell IL10 secretion at doses between 0.01-10nM by co-culture of ESC and U937 (p<0.05; **Fig 3B**). However, TCDD did not regulate IL12 secretion by macrophages co-cultured with ESCs (p>0.05;**Fig 3B**). TCDD inhibited CD86 and HLA-DR expression (p<0.01; p<0.05;**Fig 3B**). These results indicate that TCDD can induce M2 macrophage polarization during co-culture with ESCs.

### The combination of 17β-estradiol and TCDD promotes M2 macrophage polarization in the co-culture

Our previous study demonstrated that the combination of 17β-estradiol and TCDD increased RANTES secretion, a chemokine known to induce M2 macrophage activation[5–6]. To further investigate whether the combination of 17β-estradiol and TCDD regulates macrophage cytokine and surface molecule expression in the endometriotic milieu, we measured secretion of IL10, IL12 by U937 cells and expression of HLA-DR, CD86 in it. As shown in **Fig 3C**, the combination of 17β-estradiol and TCDD had synergistic effects on IL10 release by U937 cells in co-culture (P<0.05), but 17β-estradiol did not show synergy with TCDD on U937 cell IL12 secretion (**Fig 3C**. P>0.05). Compared to control, 17β-estradiol had no effect on CD86 expression by U937 cells (P>0.05). However, the combination of 17β-estradiol with TCDD had synergistic effects on CD86 expression of U937 in our co-culture system (**Fig 3C,** P<0.05;p<0.01). In co-cultured of ESCs and U937 cells, either 17β-estradiol or TCDD alone decreased U937 HLA-DR expression, and in combination, they showed synergistic effects (**Fig 3C,** P<0.05).

### The combination of 17β-estradiol and TCDD promotes M2 macrophage polarization through activating STAT3 and p38MAPK signal pathway

**Fig 4:**
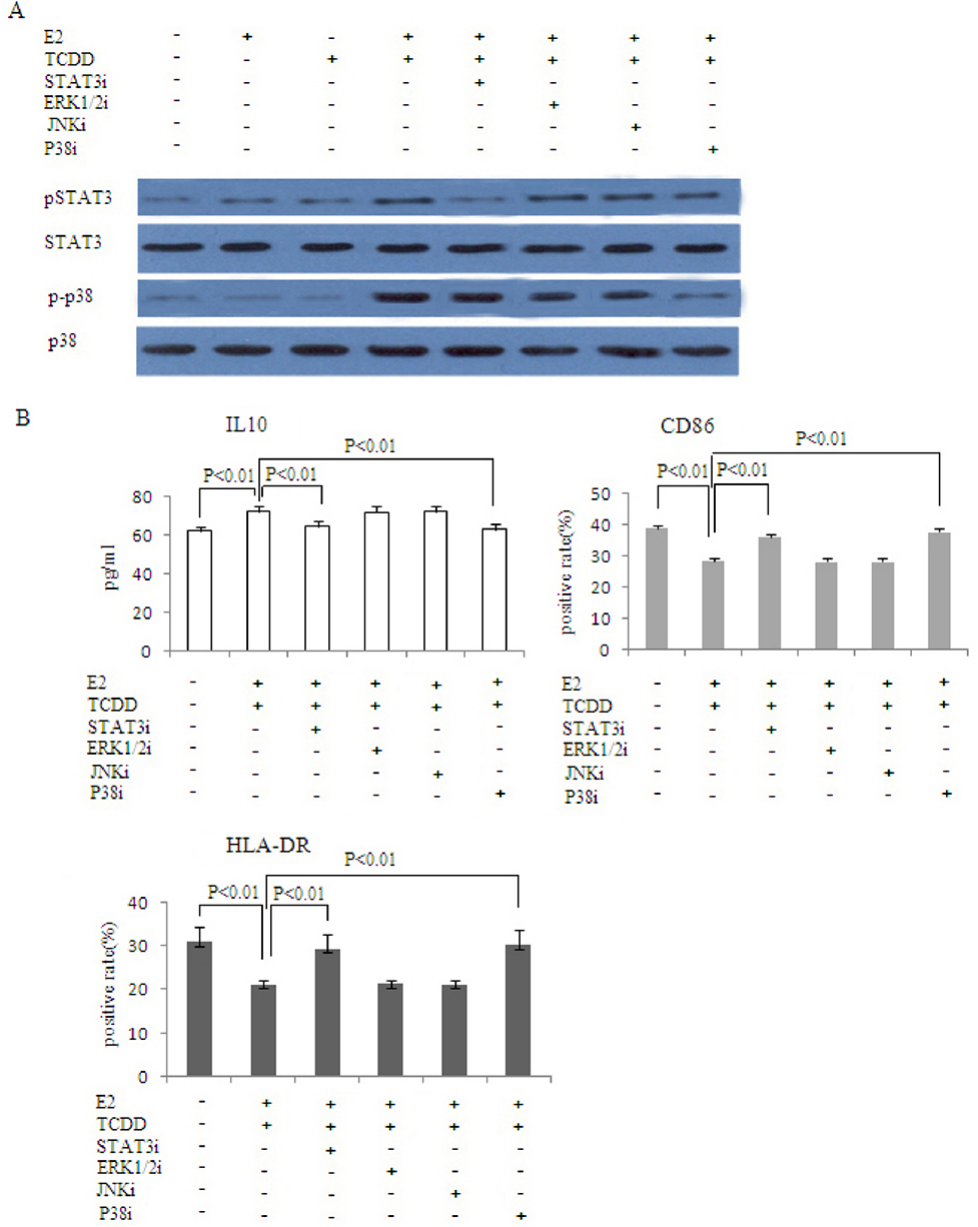
STAT3 and p38MAPK pathway are involved in the combination of 17β-estradiol and TCDD-induced M2 polarization of macrophages. Co-cultured ESC and U937 cells were incubated with 20uM STAT3 pathway inhibitor D4071, 20uM ERK1/2 pathway inhibitor U0126, 20uM JNK pathway inhibitor SP600125 or 20uM p38MAPK pathway inhibitor SB203580. After 30 minutes, 1nM 17β-estradiol and 1nM TCDD were added to the cells, with PBS serving as the control. 48 hours later, western blot was performed to analyze the levels of the phosphorylated forms and total amounts of STAT3 and P38 in U937 cells(A). IL-10 was measured by ELISA. Surface expression of CD86 and HLA-DR in U937 cells was determined by flow cytometry(B). STAT3i: STAT3 pathway inhibitor; ERK1/2i: ERK1/2 pathway inhibitor; JNKi: JNK pathway inhibitor; P38MAPKi: P38MAPK pathway inhibitor.

To determine the signal transduction pathways responsible for 17β-estradiol/TCDD- mediated M2 macrophage polarization, the co-cultured cells were treated with 17β-estradiol/ TCDD combined with different signaling pathway inhibitors (STAT3i, ERK1/2i, JNKi, P38MAPKi). In addition, STAT3, ERK1/2, JNK and P38MAPK phosphorylation were detected by immunoblotting. The combination of 17β-estradiol and TCDD induced STAT3 and P38MAPK phosphorylation in U937 cells co-cultures with ESCs (P<0.01) (**Fig 4A**). STAT3 or P38MAPK activation induced by combination of 17β-estradiol/TCDD treatment in U937 cells was blocked by STAT3 or P38MAPK inhibitor, respectively (P<0.01) (**Fig 4A**).

Since the combination of 17β-estradiol and TCDD increased IL10 secretion and decreased HLA-DR and CD86 expression of U937 in our ESC co-culture system, we asked whether STAT3 and/or P38MAPK inhibitors might influence these changes. The effect of 17β-estradiol combined with TCDD was effectively abrogated by STAT3 or P38MAPK inhibitors (P<0.01), while ERK1/2 or JNK inhibitors had no effects(P>0.05) (**Fig 4B**).

## Discussion

Estrogen is essential for the growth and maintenance of ectopic endometrial implants, and plays a major role in disease-associated biological changes and clinical manifestations[28]. TCDD can induce endometriosis[29–31] and estrogen-dependent tumors[32–33], which could be due to estrogenic effects. However, the molecular mechanisms underlying estrogen-related actions of dioxins remain largely unknown. TCDD can stimulate ERα and ERβ transcriptional activity by inducing formation of a complex between ER and ligand-bound AhR[34]. To test the hypothesis that 17β-estradiol and TCDD regulate M2 macrophage polarization, and may be a potential pathogenic mechanism for endometriosis, we treated ESCs and macrophages with a series of concentrations of estrogen and/or TCDD, and then determined the phenotypes of the macrophages. Our results suggest that M2 macrophage-related molecules, such as IL10, are significantly up-regulated by the combination of estrogen and TCDD. Estrogen alone has few effects on M2 macrophage polarization, although it facilitates the stimulatory effect of TCDD in the endometriotic milieu. Furthermore, 17ß-estradiol can activate peritoneal macrophages with respect to morphologic changes and cytokine expression, further supporting our notion that macrophages activated by 17β-estradiol might play a permissive role in the development of endometriosis[35]. Therefore, high concentrations of 17β-estradiol and TCDD in the inflammatory milieu may promote the onset and development of endometriosis via providing a ‘permissive’ immuno-inflammatory microenvironment.

Peritoneal immune surveillance systems are impaired in endometriosis[36–37]. An increased number of active macrophages have been found in peritoneal fluid of patients with endometriosis[38]. Alteration of the balance between M1 and M2 macrophages might be involved in the pathogenesis of pelvic endometriosis, which might in turn cause an increase in the local production of factors promoting angiogenesis and implantation of endometrial cells[7]. M2 macrophages possess an IL12^low^/IL10^high^ phenotype, and are generally better adapted to tissue remodeling[9]. Endometriotic tissue is composed mainly of the ectopic endometrium, macrophages and extracellular matrix. Given that retrograded ESCs are responsible for the adherence and implantation of endometrium into the peritoneum in the early stage of endometriosis, we used ESCs rather than endometrial epithelial cells for our co-culture assays to represent the retrograded endometrium[39]. In the present study, we have found that induced M2 polarization of macrophages by combination of 17β-estradiol and TCDD in the endometriotic milieu requires coordinated interaction between ESCs and macrophages. Co-culture of ESCs with U937 cells induces M2 macrophage polarization, suggesting that shed ESCs represent a foreign entity, initiating an acute inflammatory response by recruiting monocytes. Cross-talk between retrograde ESCs and macrophages ultimately induces macrophage tolerance. Our results provide new insight into the formation of M2 macrophages in the endometriotic milieu. We suspect that the regulation of macrophage M2 polarization may lead to new therapeutics for endometriosis.

Numerous in vivo and in vitro studies have reported roles of TCDD-mediated modifications of growth factor and cytokine signaling in immunosuppression. For instance, TCDD-stimulated changes in transforming growth factor (TGF)-α, TGF-β, tumor necrosis factor (TNF)-α, interleukin (IL)-1α, IL-2, IL-6, IL-8 and interferon-α gene expression have been reported[40–43]. TCDD induces functional Treg cells that suppress experimental autoimmune encephalitis[44]. IL10 is considered to be the most important anti-inflammatory mediator[45]. In our study, treatment with combination of 17ß-estradiol and TCDD increased IL10 production in macrophages when co-cultured with ESCs. Signal transducer and activator of transcription 3 (STAT3) is the key mediator of the anti-inflammatory effects of IL10[46–48]. STAT3 signaling in macrophages is well known to be involved in the regulation of immune responses in murine models[46,49], and STAT3 activation is essential for M2 macrophage differentiation[50]. The p38MAPK pathway has a key role in the regulation of the inflammatory response by orchestrating pro- and anti-inflammatory effector mechanisms. Cross-talk between p38 and AhR signaling pathways and the role of p38 in AhR signaling has been explored in several studies. Activation of p38, and possibly other MAP kinases, by AhR receptor ligands (such as TCDD) seems to be a cell type-specific consequence of ligand exposure. TCDD activates p38 and ERK1/2 in RAW 264.7 murine macrophages by an AhR-independent mechanism [51], and it also activates JNK and ERK, but not p38, in mouse embryonic fibroblasts and African Green Monkey kidney CV-1 cells [52]. In our present study, the combination of 17β-estradiol and TCDD induces M2 polarization of macrophage through STAT3 and P38MAPK activation, while the ERK1/2 and JNK pathways appear to be dispensable. STAT3 or p38MAPK inhibitors could not completely abrogate the differentiation of M2 macrophage, implying that other signaling pathways may also be involved in the combination of 17β-estradiol and TCDD-induced M2 macrophage activation (Fig 4).

These studies provide the first evidence that 17β-estradiol and TCDD coordinate to promote M2 macrophage polarization via both STAT3 and p38MAPK activation in the endometrial milieu. Future studies will be required to more fully characterize the activation state of macrophages in the endometriotic milleu. These findings provide new insights into the mechanisms of estrogen and TCDD interactions in endometriosis. We hope that our study ultimately leads to the design and development of novel therapeutic regimens for endometriosis.
